# The Relation of Hospitals to Medical Education
*By Charles Francis Withington, M.D. (Boston: Cripples, Upham, and Co.)


**Published:** 1889-02-02

**Authors:** 


### The Relation of Hospitals to Medical Education.*

It needs no argument to show that for the medical education of the individual clinical instruction is a *sine qua non.* And if medical students do not obtain such instruction in hospitals there are no other means, especially now the system of apprenticeship has been abandoned, by which neophytes can obtain the required information. Hospitals, both general and special, have the material in large amount, and Dr. Withington considers the right to use it. No doubt the primary intention of the founders of the world's great hospitals, from the middle ages down to modern times, has been the relief, during times of sickness, of those who are unable properly to provide for themselves. But there are few, if any, endowed institutions which are carried on precisely as their originators supposed they would be. Times alter in a manner which never occurred to such originators; and there is little doubt that were founders and originators still upon the stage of action, they would, in the exercise of the same judgment and generosity which characterised their lives, endorse and approve the action made necessary by changed conditions. In remote days, the great majority of students in medicine derived what clinical experience they possessed, from accompanying their masters in their daily rounds. But it would be difficult, if not impossible in the present day, to obtain the consent of those paying for their medical attendance, to allow themselves to be used for the purposes of clinical instruction. And, moreover, the instruction to be derived from the practice of one physician would be infinitely less than that obtainable from even the smallest hospital. Also it would be given only to one person, whereas in a hospital instruction is imparted to many at one time, and by one labourer. "From free patients, however," Dr. Withington remarks, "we claim such a *quid pro quo* as can be rendered without detriment to the patients," And this claim is made chiefly in the interests of the future. The only limitation to this requirement is that the facilities for instruction which are to be employed shall not be prejudicial to the recovery or welfare of the individuals. There are various appliances in hospitals peculiarly useful for observing and collecting facts of scientific value, and such appliances are more or less unattainable elsewhere. Records of cases can be kept in a hospital with a completeness possible nowhere else. The qualifications of the hospital staff are centred, and available alike for the investigation of disease and for clinical instruction. The control of conditions, nursing for instance, is much more complete in a hospital than it can be in private practice. All these are positions which render the hospital far superior as a clinical school than attendance on any physician's private practice, even if this were possible for the numerous students thronging our medical schools.

The objections which patients make are that they are subject to experiments, to over frequent physical examinations, and to *post mortem* procedure. But in the present state of medicine, any treatment adopted is to a certain extent experimental, for temperaments and idiosyncracies differ to such an extent, that even the longest used medicine may act differently on different people. The physiological action of new drugs is well ascertained by experiments on animals long before it is given to human beings, and unless there is a prospect of cure *cito tuto et jucunde,* it is not given to human beings either in or out oi hospitals. If certain operations had not been performed at some risk when first proposed, we should not now have the brilliant results, and the very large proportion of cures recorded. "Hospital patients, then, cannot complain if they have to bear a part in the solution of the medical problems of the day, in so far, at least, as a similar obligation rests upon the sick who are outside of such institutions. They have, however, a right to immunity from experiments merely as such, and outside of therapeutic application. Over-frequent physical examinations may sometimes work an injustice to hospital patients, especially repeated and prolonged examination of the chest during acute diseases of the contained viscera. This, however, is in the power of the physician to forbid, and we believe such examinations are never carried to excess in Britith hospitals. As regards *post-mortem* examinations they are never insisted upon against the expressed wish of the person during life, or of his nearest friend after death. One great dread in the minds of a portion of the public in entering a hospital is that of being dissected should they die. However we may regard this as a sentimental objection, there is no moral right to disregard it. And this, we believe, is never done.

Dr. Withington observes that there are other facts increasing the educational value of hospitals, and to this we add increasing also the curative power of hospitals. It is pointed out as a matter of frequent experience that men who have made for themselves a distinguished reputation in science or literature are by no means always the most successful teachers of the subject which they themselves have so fully mastered. But attached to hospitals and medical schools there are younger men, and many a student learns more from the adjunct professors, the instructors and the tutors in our colleges—men who are nearer the student's own age, and can better appreciate his difficulties—than from the professors, despite the possibly greater attainments of the latter. As another factor, we have resident physicians and surgeons, and there is every reason why these posts should be awarded (as we believe they are awarded) strictly on the basis of professional qualification. A large amount of information is usually imparted to students by resident physicians and surgeons, while patients have always at hand men professionally skilled both to carry out the orders of the visiting doctors and, if necessary, to act on emergency. A third factor is hospitals being a training school for nurses. The most useful handmaid of medicine is nursing. For the education of a class of persons fit to carry on this important work hospitals afford peculiar advantages. Before 1860, when Miss Nightingale established at St. Thomas's Hospital the first training school for nurses, there had been no instrumentality outside of certain religious orders for systematic and thorough instruction in nursing. The majority of nurses were "Sarah Gamps." Now nurses specially educated are obtainable. But nurses, like medical students must pass through a period of probation, and must gain their experience from hospital patients. Hospital patients are, however, better nursed than they were formerly, and by allowing probationers to nurse them, have contributed to this desirable result. Another factor is hospital reports, as for instance the reports annually published by the medical staff of Guy's Hospital, which diffuse the experience gained, both to other hospitals and to private practitioners. Thus it appears that modern hospitals must serve two purposes, viz., the cure of the sick, and medical education. No one will deny the first obligation; and as it can be shown that hospitals possess certain facilities for the advancement of medical science that are not enjoyed elsewhere, the responsibility must be met by fulfilment of the obligation.

* By Charles Francis Withington, M.D. (Boston: Cripples, Upham, and Co.)

### GOOD SERVICE.

"Thy love
Shall chant itself its own beatitudes,
After its own life-working. A child's kiss
Set on thy sighing lips shall make thee glad;
A poor man, served by thee, shall make thee rich;
An old man, helped by thee, shall make thee strong;
Thou shalt be served thyself by every sense
Of service which thou renderest."

—*E. B. Browning.*

